# Identification of host factors for livestock and poultry viruses: genome-wide screening technology based on the CRISPR system

**DOI:** 10.3389/fmicb.2024.1498641

**Published:** 2024-11-21

**Authors:** Shijie Hu, Mailin Gan, Ziang Wei, Pan Shang, Lei Song, Jinkang Feng, Lei Chen, Lili Niu, Yan Wang, Shunhua Zhang, Linyuan Shen, Li Zhu, Ye Zhao

**Affiliations:** ^1^Farm Animal Genetic Resources Exploration and Innovation Key Laboratory of Sichuan, Sichuan Agricultural University, Ya’an, China; ^2^State Key Laboratory of Swine and Poultry Breeding Industry, Sichuan Agricultural University, Ya’an, China; ^3^Key Laboratory of Livestock and Poultry Multi-Omics, Ministry of Agriculture and Rural Affairs, College of Animal and Technology, Sichuan Agricultural University, Ya’an, China

**Keywords:** CRISPR/Cas9, library screening, gene editing, viral host factors, antiviral

## Abstract

Genome-wide CRISPR library screening technology is a gene function research tool developed based on the CRISPR/Cas9 gene-editing system. The clustered regularly interspaced short palindromic repeats/CRISPR-associated genes (CRISPR/Cas) system, considered the third generation of gene editing after zinc finger nucleases (ZFN) and transcription activator-like effector nucleases (TALEN), is widely used for screening various viral host factors. CRISPR libraries are classified into three main categories based on the different functions of Cas9 enzymes: CRISPR knockout (CRISPR KO) library screening, CRISPR transcriptional activation (CRISPRa) library screening, and CRISPR transcriptional interference (CRISPRi) library screening. Recently, genome-wide CRISPR library screening technology has been used to identify host factors that interact with viruses at various stages, including adsorption, endocytosis, and replication. By specifically modulating the expression of these host factors, it becomes possible to cultivate disease-resistant varieties, establish disease models, and design and develop vaccines, among other applications. This review provides an overview of the development and technical processes of genome-wide CRISPR library screening, as well as its applications in identifying viral host factors in livestock and poultry.

## 1 Introduction

Genome-wide CRISPR library screening has been widely employed in antiviral immune response research, animal disease modeling, and drug target screening. Whole-genome CRISPR screening starts by designing an sgRNA library targeting the entire genome, which is introduced into target cells with a Cas9-expressing vector via lentiviral or other methods. After applying a selective challenge, cells from both control and challenged groups are collected. High-throughput sequencing then analyzes sgRNA enrichment or depletion, identifying host factors linked to the challenge (Shalem et al., 2015). Currently, the Genome-wide CRISPR screening technique is widely used to screen for host cytokines that interact with viral infections (Munir et al., 2024; Peng et al., 2024; Yang and Zhang, 2023). This technology can be applied to identify host factors involved in various stages of viral processes, such as adsorption, endocytosis, and replication. Subsequently, these host factors can be targeted to design antiviral drugs and vaccines, as well as to develop disease-resistant livestock and poultry breeds. This review provides a brief overview of the principles and processes of whole-genome screening technologies based on the CRISPR/Cas system, and their applications in identifying host factors of livestock and poultry viruses.

## 2 Gene editing technology development

The evolution of gene editing technologies has primarily involved the advancements of zinc finger nucleases (ZFNs), transcription activator-like effector nucleases (TALENs), and CRISPR/Cas9. All three contain a DNA-binding component that specifically recognizes and binds to certain gene fragments, as well as a nuclease component that cuts the DNA double strand. Each technology was used for gene editing during their respective periods.

### 2.1 ZFN

ZFN, developed in 1996, is composed of zinc finger proteins (ZFP) that target specific DNA sequences, and the *Fok*I restriction enzyme that cleaves DNA (Kim et al., 1996). It pioneered a new era of targeted gene editing as the first-generation gene editing tool. However, ZFN is challenging to apply in practice due to its complex and costly design and validation process.

### 2.2 TALEN

TALEN, developed in 2010, consists of transcription activator-like effector (TALE) proteins and the nuclease *FokI* (Boch et al., 2009; Christian et al., 2010; Moscou and Bogdanove, 2009). TALEN works similarly to ZFN as the second generation of gene editing tools. However, TALEN is simpler to design and construct, offering more target sites and is more stable in application. In 2012, it was recognized as one of the top ten technological breakthroughs of the year.

### 2.3 CRISPR/Cas9

CRISPR/Cas9 was officially introduced in early 2013, consists of an sgRNA that targets specific DNA sequences and the Cas9 enzyme that cleaves DNA. CRISPR/Cas systems were first discovered in *Escherichia coli* in 1987 (Ishino et al., 1987) and later linked to the immune system of the bacteria (Barrangou et al., 2007). One type of this system (CRISPR/Cas9) was adapted into a gene editing tool. Unlike the earlier systems (ZFN and TALEN), which required the construction of specific binding proteins for each editing site, the CRISPR/Cas9 system directs the Cas9 nuclease to the editing site by an approximately 20-nucleotide (nt) sgRNA that is complementary to the target sequence. CRISPR/Cas9 has accelerated the advancement of genome-wide library screening techniques due to its simple design and exceptional gene editing capabilities.

## 3 CRISPR library screening related systems

**FIGURE 1:**
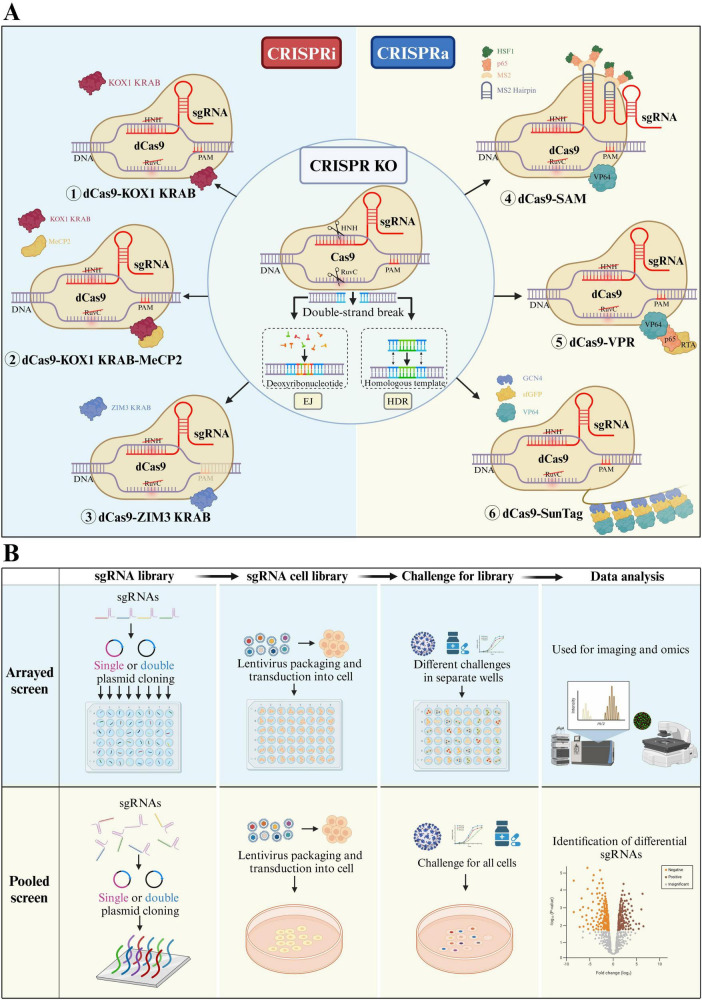
**(A)** Three types of CRISPR library screening systems: The CRISPR KO system consists of sgRNA and Cas9 protein. After the HNH and RuvC domains cleave the DNA, the broken DNA is repaired through end joining (EJ) or homology-directed repair (HDR). CRISPRi and CRISPRa systems are derived from the CRISPR KO system. In these systems, the HNH and RuvC domains of Cas9 are deactivated, resulting in dCas9, which loses its cutting ability. dCas9 forms a complex with transcriptional repression domains (①KOX1 KRAB②KOX1 KRAB-MeCP2③ZIM3 KRAB) or transcriptional activation domains (④SAM⑤VPR⑥SunTag) and combines with sgRNA to create the CRISPRi or CRISPRa systems. **(B)** Two types of CRISPR library screening workflows: Both processes mainly consist of four steps. First, sgRNA is designed and synthesized, followed by cloning the sgRNA into a single- or dual-plasmid system to create an sgRNA library. Next, the sgRNA library is introduced into cells via lentiviral transduction, knocking out the target genes. After antibiotic selection (as the sgRNA plasmid carries an antibiotic resistance gene), an sgRNA cell library is formed. Then, based on the experimental goals, selective pressure is applied to the cell library, and the surviving cells are collected. Finally, high-throughput sequencing, combined with bioinformatics analysis, is used to identify sgRNAs that are significantly enriched (positive selection) or depleted (negative selection) before and after treatment.

CRISPR screening systems are classified into two principal categories based on the different Cas enzymes employed and the resultant gene-editing effects: CRISPR knockout screening systems and CRISPR transcriptional regulation screening systems. The transcriptional regulation screening system further includes the CRISPR interference (CRISPRi) screening system and the CRISPR activation (CRISPRa) screening system (Figure 1A). Currently, all three systems are employed in library screening to study gene functions.

### 3.1 CRISPR knockout screening system

The CRISPR/Cas9 knockout system primarily consists of the Cas9 protein, which performs the cutting function, and the sgRNA, which guides the Cas9 protein to the editing site. After reaching the editing site, the two strands of DNA are cleaved by the HNH and RuvC domains of the Cas9 protein, resulting in a double-strand break (DSB). This break triggers the cell’s endogenous DNA repair mechanisms, which repair the broken DNA through end joining (EJ) or homology-directed repair (HDR). EJ is the main repair mechanism in cells following gene knockout by the CRISPR/Cas9 system (Xue and Greene, 2021). This process repairs the broken double strand by randomly recruiting deoxyribonucleotides, leading to base pair insertions or deletions that alter the original gene sequence, thereby achieving the goal of gene knockout (Nambiar et al., 2022). Genome-wide CRISPR knockdown screening creates a diverse cell library with single-gene knockouts by co-transfecting sgRNAs and Cas9 vectors into cells, which cause gene disruption and trigger the cell’s EJ repair mechanism to alter the original gene sequence.

### 3.2 CRISPR transcriptional regulatory screening system

The CRISPR transcriptional regulation system primarily consists of dead Cas9 (dCas9) that carries transcriptional regulatory elements and sgRNA that provides guidance. dCas9 is engineered from Cas9 and lacks the ability to cleave DNA due to the simultaneous inactivation of its RuvC and HNH nuclease domains. However, it can still bind to the target gene sequence under the guidance of sgRNA (Qi et al., 2013). Both CRISPRi and CRISPRa regulate transcription through the use of dCas9 combined with corresponding transcriptional interference or activators. In CRISPRi, dCas9 is combined with the transcriptional repression domain KRAB, which is guided by sgRNA to the transcription start site (TSS) of the target gene, thereby blocking transcription and inhibiting gene expression (Gilbert et al., 2013). Initially, the KOX1 KRAB domain, which was fused to dCas9, exerted its transcriptional repression function by recruiting the methyl transferase SETB1 to methylate histone H3K9 (Iyengar and Farnham, 2011). Subsequently, researchers conducted a comprehensive comparison of various KRAB domain combinations and identified that the KOX1 KRAB-MeCP2 and ZIM3 KRAB domains exhibited more pronounced inhibitory effects (Alerasool et al., 2020; Yeo et al., 2018).

Similar to CRISPRi, CRISPRa involves the combination of dCas9 with the transcriptional activation domain VPR (Perez-Pinera et al., 2013), which is guided by sgRNA to the TSS locus to facilitate transcription of the target gene. To enhance transcriptional regulation efficiency, research shows that increasing the number of transcriptional activators by combining the tripartite transcriptional activator VPR (VP64-p65-RTA) or multiple copies of the GCN4 activation recruitment domain (SunTag) with dCas9 can improve transcriptional activation efficiency (Chavez et al., 2015; Kampmann, 2018). Additionally, sgRNA can be modified by fusing it with the MS2 stem-loop, which then combines with the dCas9-VP64 fusion protein to form the SAM system. This system can also enhance transcriptional activation by recruiting the MS2-p65-HSF1 fusion protein (Konermann et al., 2015).

Currently, CRISPRi and CRISPRa library screenings are widely applied in fields such as drug target identification (Hazan et al., 2024; Kurata et al., 2018; Saito et al., 2023), drug resistance (Li et al., 2021; Poulton et al., 2024), virology (Heaton et al., 2017; Li et al., 2023), non-coding RNA (Bester et al., 2018; Halasz et al., 2024), functional genomics (Alda-Catalinas et al., 2020; Tian et al., 2019; Wang Y. et al., 2024), and receptor-ligand interactions (Yang L. et al., 2024).

## 4 Genome-wide CRISPR library screening technology process

CRISPR library screening involves two main steps: the construction of the sgRNA cell library and the screening of target factors (Shalem et al., 2014). sgRNAs can be designed with tools such as Cas-OFFinder (Bae et al., 2014), sgRNA.Scorer 2.0 (Chari et al., 2017), and CRISPR-offinder (Zhao et al., 2017). Currently, there are nearly complete libraries in various fields (Chulanov et al., 2021). The sgRNA and Cas9 plasmid usually delivered into target cells via lentivirus to construct a cell library, which can be categorized into arrayed and pooled libraries depending on the selected screening targets and focus areas (Yang C. et al., 2024). Based on different research objectives, the constructed cell library is subjected to screening factors such as drugs, viruses, harmful substances, or adverse environmental conditions. Subsequently, control cells and cells that survived under selective conditions are collected. The abundance of sgRNA in cells before and after treatment is assessed through high-throughput sequencing combined with bioinformatics analysis. Comparative analysis then identifies factors that are significantly enriched (positive selection) or significantly depleted (negative selection) (Figure 1B).

## 5 CRISPR library screening for host factor identification in livestock and poultry viruses

Viral diseases in livestock and poultry are major factors affecting economic production. The complex interactions between viruses and host cells complicate disease treatment, making the identification of host factors involved in viral interactions a critical challenge for precision therapeutics. With advancements in CRISPR gene-editing technology, CRISPR library screening has been applied to identify key antiviral host factors in livestock and poultry. Studies have confirmed that CRISPR library screening can successfully identify host factors that interact with viruses at multiple stages, including adsorption, endocytosis, and replication (Table 1). These host factors provide valuable insights into the molecular-level prevention and treatment of viral diseases.

**TABLE 1:** Identify host factors for livestock and poultry viruses using CRISPR library screening.

Virus	Susceptible animals	Cell line	Gene count	Candidate factor	Main factor	Effect	References
EEEV	Horse	N2a	19,050	Sema5a, Olfr970, Ldlr, Ccp110, Svs2	LDLR	Adsorption	Ma et al., 2024
PDCoV	Porcine	LLC-PK	20,081	TMEM41B, SLC35A1, SNX10, VOPP1, PCSK6	SLC35A1	Adsorption	Wang et al., 2022
H5N1	Poultry	A549	19,050	SLC35A1, CIC, IRX3, C2CD4C, TRIM23, PIGN	SLC35A1	Adsorption	Han et al., 2018
PEDV	Porcine	Vero E6	18,993	SLC35A1, TRIM2, PSD3, CENPQ, SLC7A11	SLC35A1, TRIM2	Adsorption	Wang et al., 2023
SBV	Ruminant	HEK-293T	19,114	SLC35B2, OR5P3, PLEKHS1	SLC35B2	Adsorption	Thamamongood et al., 2020
JEV	Porcine	PK15	17,743	B3GAT3, GLCE, HS6ST1, SLC35B2	B3GAT3, GLCE, HS6ST1, SLC35B2	Adsorption	Zhao et al., 2020
PR8	Poultry	A549	18,865	B4GALNT2, RIN2, TM9SF2	B4GALNT2	Adsorption	Heaton et al., 2017
PRV	Porcine	PK15	20,598	SGMS1	SGMS1	Endocytosis	Holper et al., 2021
PEDV	Porcine	HEK-293T	20,914	OR52M1, GP1, MGEA5, ELTD1, PCKθ	PKCθ	Endocytosis	Zhou et al., 2024
H5N1	Poultry	A549	19,050	SLC35A1, IGDCC4, ZFAT, PACSIN2, ZNF471, H5N1	IGDCC4	Endocytosis	Song et al., 2021
VV	Cow	Hela	19,050	B2M, ITGB1, ACTG1, CSK, GMEMB1, GIT1, PTPN12, TAOK1	B2M	Endocytosis	Matia et al., 2022
WSN	Porcine	PK15 and CΔ2	13,735	COG8, TNK2, PPP1R9A, RPS11, ZNF263, SLC25A28	COG8	Endocytosis	Zhou et al., 2021
H7N9	Poultry	A549	19,050	CYTH2, TTC24, NANS	CYTH2	Endocytosis	Yi et al., 2022
TEGV	Porcine	PK-15	17,743	FUT8, TMEM41B, DYRK1A, MLST8, B4GAT1	TMEM41B	Replication	Sun et al., 2021
TEGV	Porcine	PK-15	17,743	DYRK1A, FUT8, DYRK1A, MLST8, B4GAT1	DYRK1A	Replication	Fu et al., 2024
PRRSV	Porcine	PK-15	13,720	KXD1, PSMD3, LGALS2, UBB, F11R, VTN	KXD1	Replication	Jiang et al., 2022
JEV	Porcine	A549	19,050	EPHA2, NF2, TAAR5, ACSL3, COPG2, ITGB1BP2	PRLHR	Replication	Liu et al., 2024
BoHV-1	Cow	MDBK	21,165	FBXW11, TFDP1, CDC45, MCM2, VIPAS39, VPS16, VPS18, GARP, EARP	GARP, EARP	Replication	Tan et al., 2023
SAPS-Cov	Porcine	Hela	19,050	ZD17, MYB, EFNA3, JPH3	ZD17	Replication	Luo et al., 2021
PRRSV	Porcine	3D4/21	–	GPS2, MGAT5B, KCNC2, SMPDL3B, FGF10	SMPDL3B	Replication	Shen et al., 2023
ASFV	Porcine	WSL	20,598	SLA-DM, SLA-DMA, SLA-DMB, RFXAP, CIITA	SLA-DM	Antiviral response	Pannhorst et al., 2023
RV	Porcine	Hela	19,050	SLC35A1, GNE, CMAS, UGCG, FA2H, LATS2	STAG2	Antiviral response	Ding et al., 2018
FMDV	Porcine	IBRS-2	16,886	TOB1, PLTP, FAM83D	TOB1	Antiviral response	Peng et al., 2024
PR8	Poultry	HeLa	18,885	B4GALNT2, ATP6V1C1, B3GAT1, JADE3	JADE3	Antiviral response	Munir et al., 2024
VSV	Porcine	Hela	19,050	TOR4A, RDH12, DNLZ, SNX18, SLC25A23, STON2, HIST1H2AH, C10orf120, GATA4	SLC25A23	Antiviral response	Zhang et al., 2023
PDCOv	Porcine	LLC-PK	–	CPSF2, HSP90AB1, SLC27A4,CCDC124, TOMM6, EDIL3, SLC9A5, DCTN1	HSP90AB1	Antiviral response	Zhao et al., 2024

−: Number of genes unknown.

### 5.1 Host factor in the virus adsorption phase

Viral adsorption to host cells is the first critical step in entry, which is driven by interactions between viral particles and specific cell surface receptors. Genome-wide CRISPR library screening has proven effective in identifying these key receptors and host factors involved in viral adsorption. The low-density lipoprotein receptor (LDLR) has been recognized as a receptor for various alphaviruses (Zhai et al., 2024). A study demonstrated that LDLR can serve as a low-affinity receptor for EEEV through CRISPR knockout screening, with the virus binding to cells by interacting with the LA domain of LDLR (Ma et al., 2024). Sialic acid receptors (SA) are carbohydrate molecules widely distributed on the surface of host cells and have been identified as specific receptors for various animal and human viruses (Everest et al., 2022). Solute carrier family 35 member A1(SLC35A1), a CMP-sialic acid transporter (cytidine 5′-monophosphate), participates in the synthesis of sialic acid receptors on the cell surface. CRISPR library screening identified SLC35A1 as a host factor affecting the adsorption of various viruses (Han et al., 2018; Wang et al., 2023, 2022). Similarly, studies have revealed that host factors involved in membrane function regulation, such as solute carrier family 35 member B2 (SLC35B2) and heparan sulfate proteoglycan pathway-related genes (HSPGs), also influence viral adsorption (Thamamongood et al., 2020; Zhao et al., 2020). Additionally, CRISPR activation library screening has revealed that β-1,4-N-acetylgalactosaminyl-transferase 2 (B4GALNT2) can reduce viral adsorption by competing with the virus for binding to sialic acid receptors (Heaton et al., 2017). Thus, the use of CRISPR library screening can identify host factors that act as viral receptors, aid in receptor synthesis, or compete with the virus for receptor binding.

### 5.2 Host factor in the endocytosis phase of viruses

Viral endocytosis is the second step in a virus’s infection of host cells, primarily dependent on interactions between viral proteins and host cell factors, and involves various intracellular biological processes. By identifying the genes associated with viral infection through CRISPR library screening, viral entry and intracellular transport can be disrupted by regulating gene expression, thereby inhibiting infection. Studies have identified genes such as sphingomyelin synthase 1 (SMS1) and Immunoglobulin Superfamily DCC Subclass Member 4 (IGDCC4), which regulate transmembrane transport, as host factors that facilitate the internalization of various viruses. Knockout of these genes significantly reduces intracellular viral load (Holper et al., 2021; Matia et al., 2022; Song et al., 2021). Additionally, CRISPR library screening has revealed that oligomeric Golgi complex 8 (COG8) and guanine nucleotide exchange factor cytohesin 2 (CYTH2), which regulate intracellular vesicle transport, not only promote influenza virus internalization but also assist in the transport of viral components between cellular compartments, thereby enhancing viral infection (Yi et al., 2022; Zhou et al., 2021). Protein kinase Cθ (PKCθ), a specific member of the protein kinase C family, primarily functions in immune and inflammatory processes. However, a study discovered that PKCθ is also involved in the internalization of porcine epidemic diarrhea virus (PEDV) and can promote viral infection through the PKCθ-BOK-caspase3 mitochondrial apoptosis pathway (Zhou et al., 2024). Accordingly, CRISPR library screening can also identify host factors involved in viral internalization and intracellular transport.

### 5.3 Host factor for viral replication stages

Viral replication is dependent on the cell’s biological machinery, with numerous cellular factors involved in this process. Genome-wide library technology can be used to identify factors that significantly impact viral replication. CRISPR library screening has identified that transmembrane protein 41B (TMEM41B), the endosome-associated recycling protein (EARP) complex and the golgi-associated retrograde protein (GARP) complex, which are involved in intracellular membrane regulation, can influence viral replication. TMEM41B facilitates the formation of the double-membrane vesicle (DMV) structures essential for the replication of the transmissible gastroenteritis virus (TEGV), thereby promoting the replication and transcription of the viral genome. GARP and EARP assist in the assembly of the envelope protein VP8 of Bovine herpesvirus 1 (BoHV-1) (Fu et al., 2024; Sun et al., 2021; Tan et al., 2023). There is a close relationship between autophagy and lipid metabolism, both of which significantly impact viral replication (Wang et al., 2019). Several studies have demonstrated that genes such as KXD1 and SMPDL3B promote the replication of viruses like porcine reproductive and respiratory syndrome virus (PRRSV) by enhancing autophagy or lipid degradation. In cells lacking these genes, viral replication is inhibited (Jiang et al., 2022; Liu et al., 2024; Shen et al., 2023; Yang et al., 2012). Additionally, zinc finger DHHC-type palmitoyltransferase 17 (ZD17) has been shown to facilitate the replication of SADS-CoV, and inhibition of its palmitoylation activity results in suppressed viral replication (Luo et al., 2021). Therefore, CRISPR library screening technology can be employed to identify host factors that influence viral genome replication or assembly.

### 5.4 Host factor in cellular antiviral responses

Viral infections trigger immune responses, while viruses counteract these defenses through evasion mechanisms. CRISPR library screening helps identify key factors in the antiviral immune response. The interferon system is a critical components of the antiviral response. Upon recognizing a viral infection, the body activates the interferon pathway, stimulating interferon production and inducing an antiviral state. On one hand, interferons limit viral spread by inhibiting viral replication or promoting cell apoptosis; on the other hand, they enhance immune responses against viral infection by promoting antigen presentation (Chiang and Liu, 2018; Schneider et al., 2014). The cGAS-STING signaling pathway plays a vital role in type I interferon (IFN-I) production. cGAS recognizes cytosolic DNA and activates STING, which moves to the Golgi apparatus and triggers the JAK-STAT and NF-κB signaling cascades, promoting IFN production (Wang and Fish, 2019). CRISPR screening has identified stromal antigen 2 (STAG2) as a factor involved in maintaining genomic stability. Its loss leads to DNA damage and an increase in cytoplasmic DNA, thereby activating the cGAS pathway to counteract infections by various rotaviruses (RV), vesicular stomatitis virus (VSV), chikungunya virus (CHIKV), and two strains of influenza A virus (Ding et al., 2018). Additionally, CRISPR screening identified jade family PHD zinc finger 3 (JADE3) and Transducer of ERBB2.1 (TOB1) as host factors that influence viral infection. JADE3 activates the NF-κB signaling pathway, promoting the production of the antiviral protein IFITM3, thereby inhibiting influenza virus infection (Munir et al., 2024). In contrast, TOB1 suppresses the phosphorylation of STATs by JAKs, inhibiting downstream interferon-stimulated gene (ISG) expression and facilitating foot-and-mouth disease virus (FMDV) infection (Peng et al., 2024). Mitochondrial antiviral signaling protein (MAVS) represents another key pathway involved in IFN-I production. After viral recognition by RIG-I-like receptors (RLRs), the MAVS pathway is activated to promote IFN-I expression (Wang J. et al., 2024). CRISPR library screening identified SLC25A23, a mitochondrial membrane adenine nucleotide transporter, which interacts with Trim31, to prevent the ubiquitination and activation of MAVS. This inhibits downstream IFN-I activation, assisting in vesicular stomatitis virus (VSV) infection (Zhang et al., 2023).

Moreover, viruses can evade host immune defenses to promote infection. Heat shock protein 90α family class B member 1 (HSP90AB1) plays roles in protein regulation and signal pathway modulation. A study found that HSP90AB1 interacts with the C-tail domain of the nucleocapsid (N) protein of porcine deltacoronavirus (PDCoV), preventing its degradation through the proteasome pathway (Zhao et al., 2024). Research in wild boar lung (WSL) cells has also demonstrated that the deletion of genes related to non-classical class II porcine leukocyte antigen DM (SLA-DM), which are involved in immune regulation, inhibits different strains of African swine fever virus (ASFV), suggesting that these genes assist in viral immune evasion (Pannhorst et al., 2023). Thus, CRISPR library screening technology can be utilized to identify host factors that either combat viral infections or assist in viral immune evasion.

## 6 Summary and prospect

In conclusion, cellular factors in host cells that interact with viruses at various stages can be identified by utilizing an sgRNA library and Cas9 enzymes with distinct functions. In fact, the process by which certain viruses infect different hosts is similar, and different species may share the similar host factors as viral receptors. For example, SLC35A1 serves as a receptor for viral attachment in both swine and poultry (Han et al., 2018; Wang et al., 2022). Conversely, antiviral host factors show significant species-specific differences among various livestock and poultry breeds. Studies have shown that pigs with a knockout of interferon-stimulated gene 15 (ISG15) are more susceptible to pseudorabies virus, whereas susceptibility to the virus is not increased in other species (Dzimianski et al., 2019; Liu et al., 2022).

Additionally, CRISPR library screening is primarily applied in livestock and poultry for identifying virus-host interaction factors, with applications in breeding disease-resistant varieties, establishing disease models, and designing vaccines. Currently, the most advanced development is PRRS-resistant pigs, which are now available commercially (Burger et al., 2024). Research on other antiviral livestock and poultry is also making progress (Idoko-Akoh et al., 2023; Li et al., 2024). Vaccine development leverages insights into viral infection mechanisms and employs gene editing to adjust viral virulence, as seen in the African swine fever vaccine (Bhujbal et al., 2022). Animal disease models are primarily established by knocking out virus-resistance genes to create disease-susceptible models, enabling studies of pathogenesis (Lin et al., 2022; Shrock and Guell, 2017). Moreover, as technology advances, related applications will continue to expand.
